# “Intracascaral space” an eggshell structure of *Caiman latirostris* eggs

**DOI:** 10.1038/s41598-021-85113-9

**Published:** 2021-03-10

**Authors:** Mila V. Piazza, Mariela S. Fernández, Pamela M. L. Leiva, Carlos I. Piña, Melina S. Simoncini

**Affiliations:** 1 Facultad de Ciencias Agrarias, Universidad Nacional de Rosario, Campo Experimental Villarino C.C. 14, Zavalla, Santa Fe Argentina; 2 Instituto de Investigaciones en Biodiversidad y Medioambiente-Consejo Nacional de Investigaciones Científicas y Técnicas, Quintral 1250, 8300 Bariloche, Río Negro Argentina; 3 Centro de Investigación Científica y de Transferencia Tecnológica a la Producción-Consejo Nacional de Investigaciones Científicas y Técnicas, España 149, 3105 Diamante, Entre Ríos Argentina; 4 Proyecto Yacaré, Laboratorio de Zoología Aplicada: Anexo Vertebrados (Facultad de Humanidades y Ciencias - Universidad de Nacional del Litoral / Ministerio de Aguas, Servicios Públicos y Medio Ambiente), Aristóbulo del Valle 8700, 3000 Santa Fe, Santa Fe Argentina; 5 Facultad de Ciencia y Tecnología, Universidad Autónoma de Entre Ríos, Tratado del Pilar 314, 3105 Diamante, Entre Ríos Argentina

**Keywords:** Electron microscopy, Zoology

## Abstract

In recent decades, eggshells of eggs from large-bodied reptiles have been studied by many researchers, to describe the eggshell, to compare them to extinct lineages that once inhabited our planet and also to understand how the egg provides the embryo specific conditions during incubation. In previous studies we described and characterized normal and pathologic *Caiman latirostris* eggshells; we also evaluated how the eggshell changes during incubation. In a study relating temperature variation and eggshell structures of successful eggs, we observed empty structures not previously described that we termed “intracascaral space”. The aim of this study is to describe this structure of *C. latirostris* eggshells. We hypothesize about the possible functions which it would perform during incubation and for development of the embryos.

## Introduction

During incubation, reptile eggshells provide mechanical protection, gas and water exchange, and calcium for embryonic development^1–4^. Within the order Crocodylia, eggs may have smooth, ornate, eroded or scaly surfaces and they can vary in degree of calcification^5,6^. In *Caiman latirostris*, eggshells are composed from the outside by their ornamentation and followed by the shell itself. According to Fernández et al.^6^ *C. latirostris* eggs are opaque and have an ornamented external surface, with lacunae, tubercles and bridges built up from sharply separated and superimposed calcareous layers. These authors^6^ also mentioned that ornamentation is columnar in microstructure, giving the shell surface its characteristic; and columns are formed by concentric deposits of calcite that are expanded at different heights and connected to one another irregularly via expansions of other adjacent columns. In brief, the shell is composed of a rigid layer made up of calcite sheets with ornamentations, which internally is interrupted by pore-canals. Towards the interior of the egg we find the inner eggshell membrane made up of a network of protein fibres, through which gases pass by diffusion^7^. *Caiman latirostris* eggs may vary in calcification, being heavily or slightly calcified^6^, and this depends on the food resources available to females^8^, and whether they live in the wild or in captivity. Calcium deficiencies in diets of captive breeding females have been reported to result in low-calcium eggs or weak shells^6^. As a result, these eggs are more vulnerable to infection by microorganisms, because shells break easily allowing pathogens such as fungi and bacteria to enter, resulting in embryo death. On the other hand, eggs that are heavily calcified could present some difficulty for embryo to hatch^9^, although in *C. latirostris* it was observed that attrition of the shell produced during incubation would reduce this effect^10^.

A peculiarity of *C. latirostris* eggshells is that they have external ornamentation such as bridges, ponds and tubers, made up of columnar structures. These are formed by concentric deposit of calcium layers with different heights, joining each other in an irregular way forming bridges. The calcite layer is composed of rhomboidal calcite crystals. Among crystals we can find what is called a “surface defect” in which a piece can be missing and from which crystals start to degrade by acid erosion. Degradation of continuous layer^11^ is faster than the rest, thus they begin to form staggered craters, which usually coincide with pores^7,12,13^. While the testaceous membrane is made up of two parts, the innermost part is made up of randomly arranged fibres, and the outer part is amorphous, with no fibrous structure^6^.

Eggshell structures are altered during incubation, due to both external and internal factors which cause an increase in the number and size of pores, and even a decrease in eggshell thickness^7,14^. Internal factors such as calcium mobilization from eggshell to embryo, allows the incorporation of this mineral during the period of bone formation, reducing thickness of the layer of the entire shell^1,5^. The presence of embryo (fertile egg) or it absence (infertile egg) affects eggshell structures differently^9^. On the other hand, the rate of embryonic development is temperature dependant, so in this aspect, environmental temperature is perhaps the most pervasive of environmental influences on reptilian development because it determines the limits to development and its rate^15^. Temperature in the nest may affects the rate of calcium mobilization. The mobilization of calcium is not only from eggshell, but also from yolk, varying the proportion of consumption according to the species, with crocodiles and birds consuming more calcium from eggshell; and some snakes consuming more from yolk^4,16^. Studies on *Alligator mississippiensis* reveal that during the third week of incubation there is a reduction in the small calcium crystals in eggshell, which appear to be mobilized in the embryo calcification^17^. In this regard, is important to test if environmental temperature affects the rate of calcium mobilization.

Among the external factors, incubation medium and fermentation of organic matter that makes up the nest material could affect the morphology and structure of eggshell^5,18^. Nest material performs important functions such as: mechanical protection, buffering sudden changes in environmental temperature. These conditions produce a suitable environment for the development of a microflora/fauna that results in fermentation of organic material, which will transfer heat to the developing eggs. Additionally, these microorganisms and their metabolic products (such as acids) degrade the outer shell, facilitating hatching upon completion of embryo development^10,19^. Microorganisms produce metabolic acids as a fermentation product of nest vegetation which in combination with carbonic acid (formed by the hydration of the expired CO_2_) dissolves calcite crystals forming craters^12^. Temperature could be another external factor that would modify the calcareous layer of eggshells, by affecting composition and density of microorganisms in the nest material, which could increase or decrease transferred heat to embryo during incubation, resulting in an increase or decrease in embryo metabolism. In addition, if metabolism of the developing embryos is accelerated or decelerated by incubation temperature, it would be expected that eggshells structures would be modified; for example, attrition of calcium layers (both externally and internally) would be greater due to the increased metabolic rate of developing embryo^20–22^. Therefore, the objective of this work was to observe how constant and fluctuating temperatures affect eggshell structures at the end of incubation.

## Methods

### Collection of samples

Five nests of *C. latirostris* were evaluated in this study, two of them coming from captive animals from the breeding facility of the Zoological Experimental Station “Granja La Esmeralda” located in Santa Fe city (Santa Fe province, Argentina). The three remaining nests were collected from Cacique Ariacaiquin (30° 39ʹ 50ʹʹ S 60° 14ʹ 05ʹʹ W) and Los Saladillos (30° 43ʹ 35ʹʹ S 60° 17ʹ 27ʹʹ W) in Santa Fe province, Argentina in December 2018, as part of the ranching program ‘Proyecto Yacaré’ (conservation and sustainable use program). Nests were located by active search by researchers within swamps on floating vegetation and forest. These five nests had an average of 32 (range 25–43) eggs which is within the normal range for *C. latirostris* clutch size^23^. At the time of harvest (both captive and wild nests), we evaluated egg viability based on the presence of opaque band^24,25^. Eggs were marked in nest to preserve their orientation to avoid embryonic death, and then removed from nest and transported in containers (plastic tank of 20 l) to Proyecto Yacaré facilities (Laboratorio de ZoologíaAplicada: AnexoVertebrados-FHUC; UNL/MASPyMA). In the laboratory, one egg per clutch was opened to determine developmental stage; the eggs had 48 h of oviposition or less (embryo developmental stage two, according to Iungman et al.^24^). Each nest was randomly divided in 4 temperature regimes and their corresponding replicas. Nests were incubated in I290 Ingelab incubators in different temperature regimes: temperatures producing only males (33 °C), only females (31 °C), and temperature fluctuations producing males and females (33 °C for 9 h and at 31 °C 15 h, and 33 °C for 15 h and at 31 °C 9 h); in order to evaluate the effect of temperature on eggshell modifications. Previous studies reported effects in some hatchlings characteristics, from different incubation constant temperatures and fluctuation incubation temperatures^26,27^. If incubation temperatures (constants and fluctuations) affected mass, size, hatchling success, it is logical to think that temperatures could affect the eggshells structures (inside and outside).

### Eggshell analysis

Once caiman hatched, we separated the inner eggshell membrane from the actual calcified eggshell. We placed eggshells in hermetically sealed bags with labels, they were not rinsed with any substance that could modify their structure, and in the case that part of the nest material (soil or plant material) was present, it was removed manually or with a very fine brush with very soft movements. We studied 100 eggs, 25 eggs per temperature treatment. From each egg we took 2 samples, one from a pole and other from the equator region, because in a previous study we reported differences in the modification of the eggshell structures between the two regions, during the development of the incubation^7^. Without any additional treatment, we observed these 200 samples with a binocular magnifying glass with a millimetre lens and measured thickness with and without ornamentation, and counted number of pores per area unit (pore density) of each sample. We selected 14 eggs (representing all nests) and observed polar and equatorial region, both in radial and top view in a SEM Phenom PRO (Scanning Electron Microscope at Electronic Microscopy Laboratory, CICyTTP/CONICET-Prov. ER-UADER). We have analysed with a General linear mixed models in which the independent variables were incubation temperature treatment, and as random variable the nest of origin, and the dependent variables were: eggshell thickness with ornamentation (total height) or without ornamentation, and pore density.

## Results

### Qualitative description of the eggshell of *C. latirostris*

**Figure 1:**
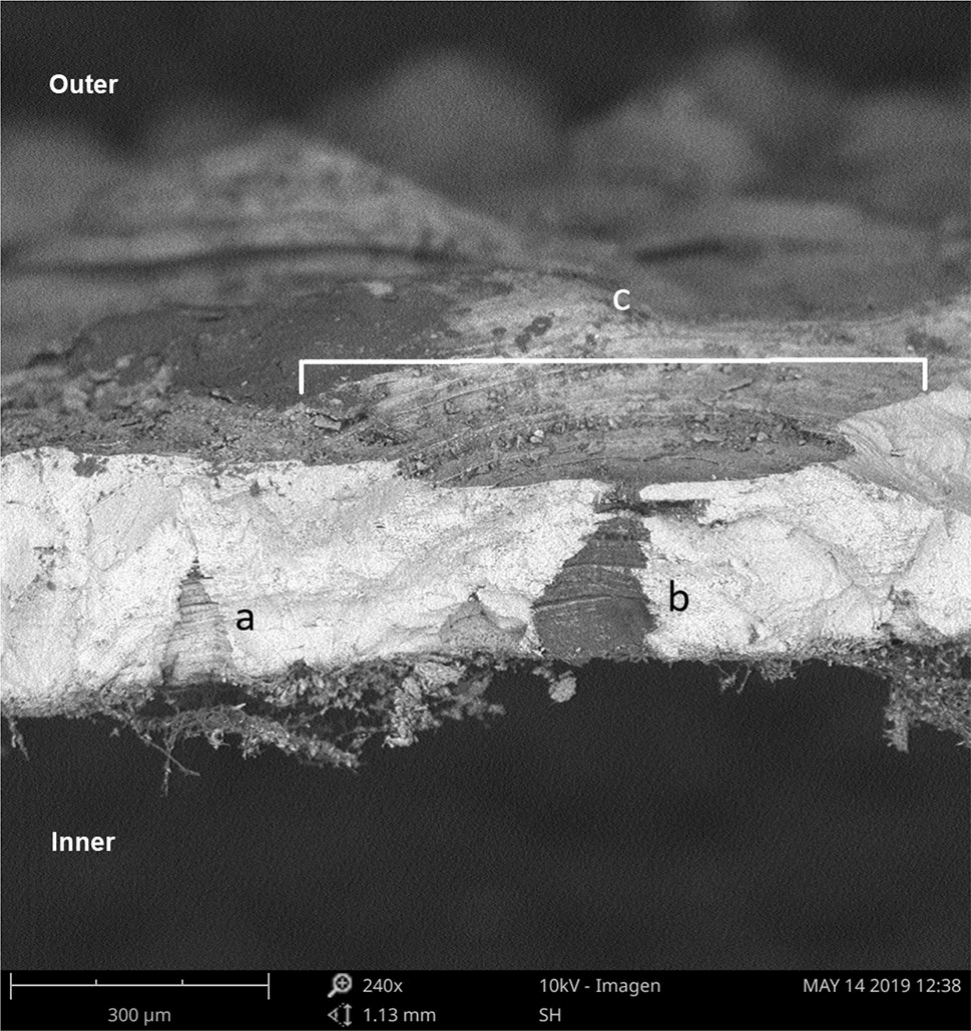
Radial view of an eggshell under SEM (Scanning Electron Microscope). We show (a) a pore-canal without external aperture, (b) pore-canal with external aperture and the air chamber in upper position, and (c) a crater above the pore aperture.

**Figure 2:**
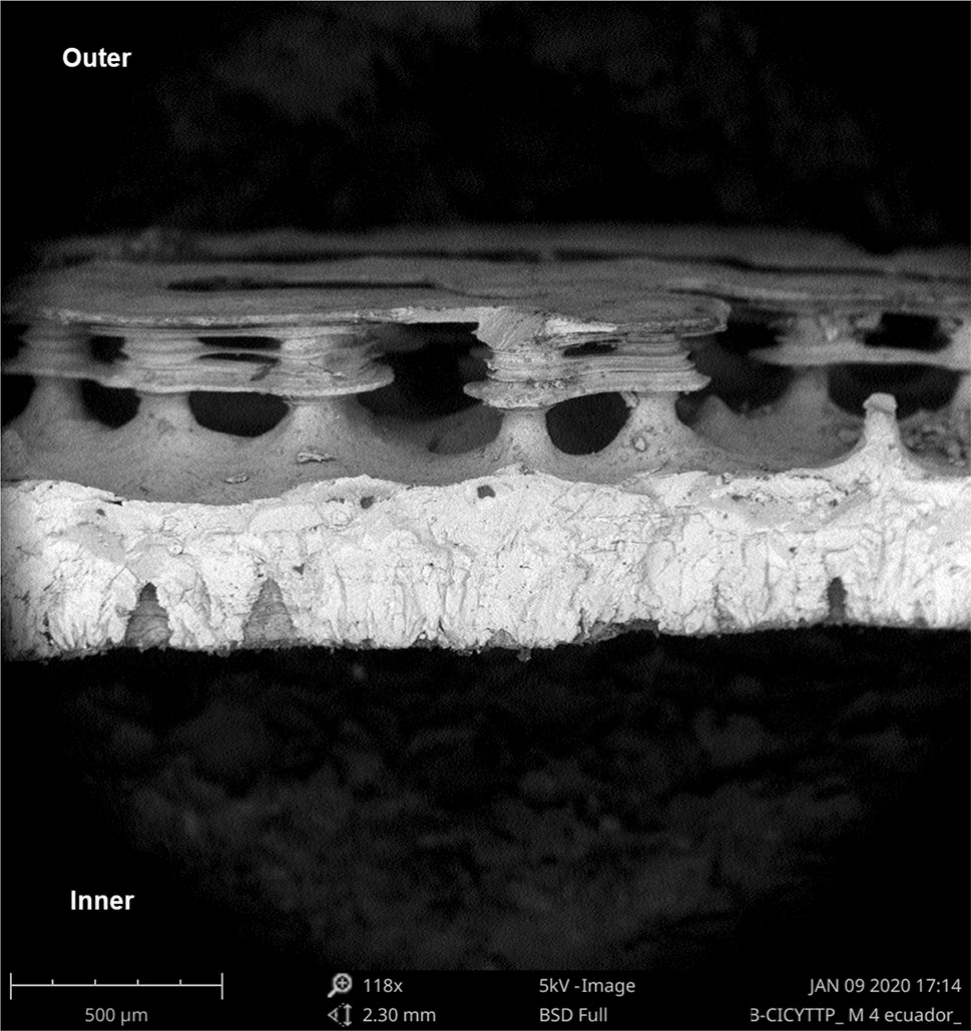
Radial view of an eggshell under SEM (Scanning Electron Microscope). This picture shows the external ornamentation form by columns and bridges between them and the main eggshell beneath with cone shaped pores. Note the absence of pore apertures in the external face.

Eggshells under SEM (Scanning Electron Microscope) view presented remains of the internal testaceous membrane adhered to the rigid layer of continuous calcite, which on its external surface showed a particular ornamentation, consisting of calcium columns (some columns joined at the top). In addition, we found channels of pores that crossed eggshells, of conical forms whose greater base was oriented towards the interior of eggshell and the vertex towards the outside, these channels connect the inside with the outside of the egg (Fig. 1). Columns had a cylindrical shape with eroded curves, ending in a head, which may be eroded to a more spherical shape or mushroom shape. These columns, in turn, could be joined to form bridges which could be described as a winding path if viewed from a top view (Fig. 2).

**Figure 3:**
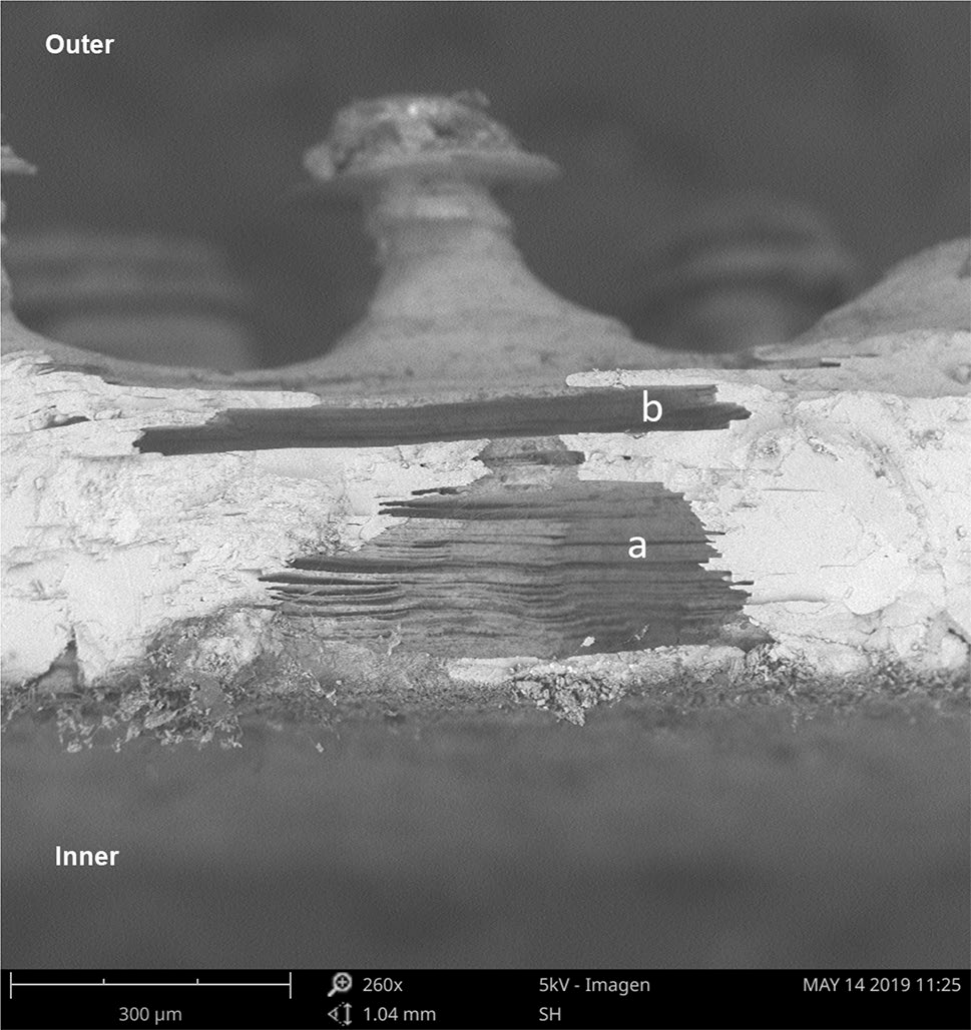
Radial view of the eggshell showing in detail the air chamber under SEM (Scanning Electron Microscope). (a) The cone of the pore canal growing to the external surface and connected with (b) the intracascaral space.

**Figure 4:**
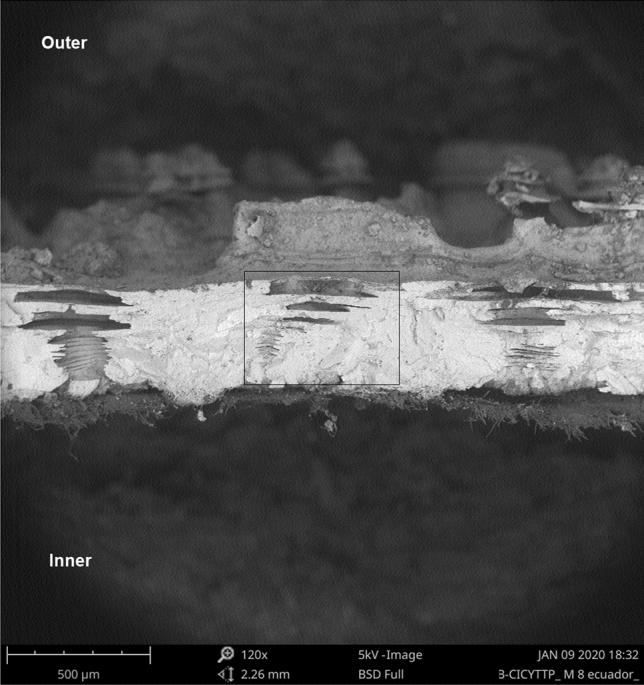
Radial view of an eggshell under SEM (Scanning Electron Microscope) showing a multiplex intracascaral spaces above the cone of the pore-canal.

**Figure 5:**
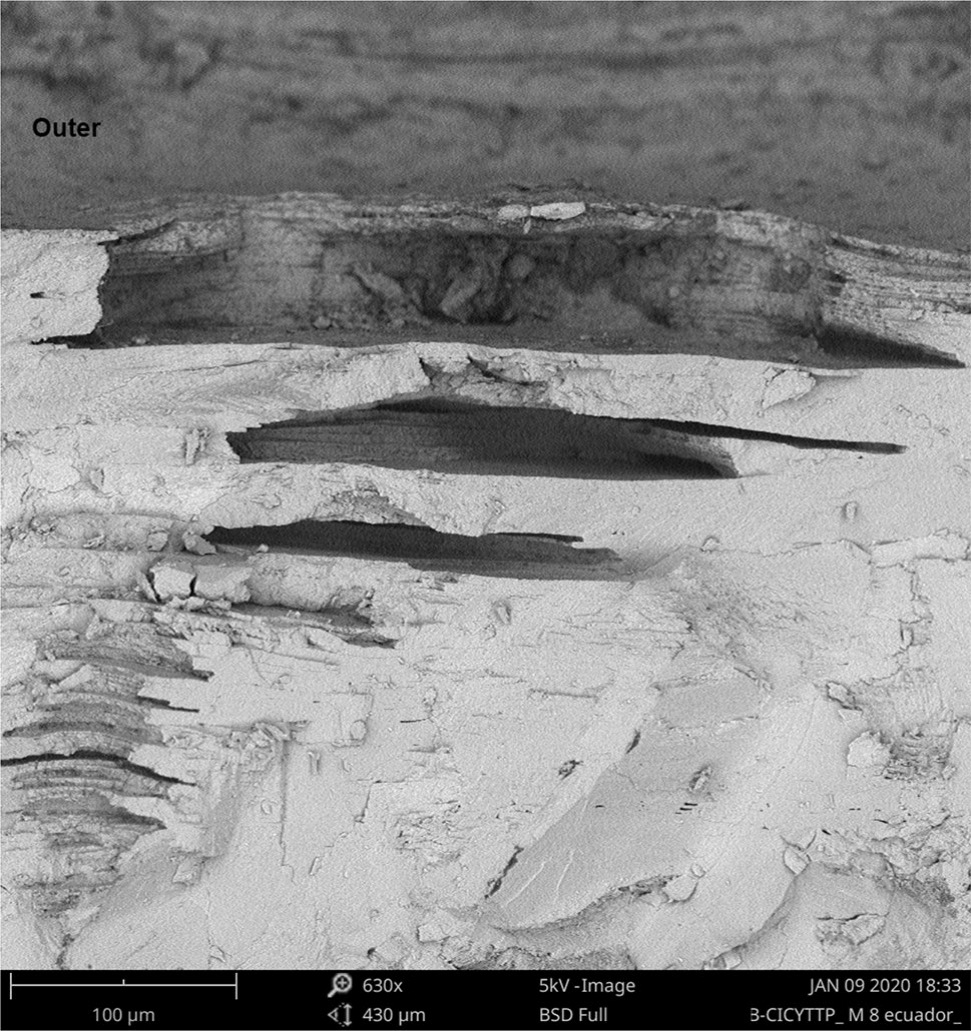
Detail of multiplex intracascaral spaces.

**Figure 6:**
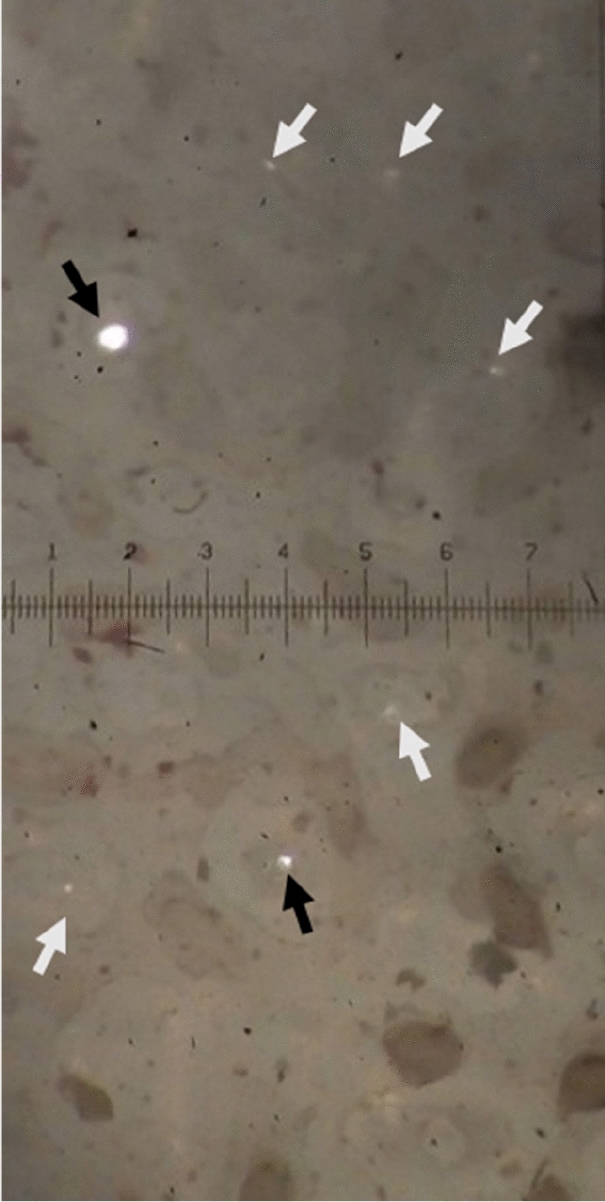
Eggshell displayed under magnifying glass (50×) in top view in backlight. Open pores are indicated by black arrows; closed pores with white arrows. The dark spots are the columns that can be joined to form bridges.

Eggs from captivity and the wild, both in the pole and equator region, presented structures not described for crocodilians in the literature, which we call “intracascaral space” (Fig. 3). The presence of these structures and their frequency are related to pore density, since they were always linked to them (whether the pore was closed or open). We found one, or up to three (only in one sample) of these empty areas) (Figs. 4, 5), elongated horizontally where calcite was absent, and were located immediately above the apex of the pores. The pore is growing outwards or eroding until it meets one of these chambers, with which it merges, opening up to the outside. Using a binocular magnifier, we were able to observe a dim light when the camera has no exit to the outside or the pore remains closed, while the light passes directly when the pore is perforated (Fig. 6).

To corroborate the presence of these new structures, we also observed under SEM eggshells from infertile eggs and in early stages caiman eggs (until 5 days of incubation), and we found “intracascaral space” in both regions (pole and equator). We did not visualize the entire intracascaral space in two samples observed, we consider that it is not that the structure is not found, but that the cut does not allow to detect it completely (because the cut is not straight, or it was made behind or in front of the structure).

### Quantitative description of the eggshell of *C. latirostris*

Incubation temperature treatments did not affect the calcareous layer structures (thickness with and without ornamentation, or pore density) of the shells of the hatched *C. latirostris* eggs (p > 0.205) (Tables 1, 2).

**Table 1:** General linear mixed models (MGLM), in which the independent variables were incubation temperature treatments, and as random variable the origin nest, and the dependent variables were: shell thickness with ornamentation (total height), thickness without ornamentation, and pore density (mean ± standard deviation).

Model	LRT	P (chi)
**Temperature treatments**		
Pore density of pole-treatment + (1/nest)	2.91	0.405
Pore density of equator-treatment + (1/nest)	4.57	0.206
Thickness with ornamentation of pole-treatment + (1/nest)	4.58	0.205
Thickness without ornamentation of pole-treatment + (1/nest)	1.88	0.598
Thickness with ornamentation of equator-treatment + (1/nest)	4.38	0.223
Thickness without ornamentation of equator-treatment + (1/nest)	2.62	0.454

Degrees of freedom of the analyses were 3.

**Table 2:** Thickness (mm) with and without ornamentations for the pole and the equator, and pore density (pores/mm^2^) for the different temperature treatments (mean ± standard deviation).

Treatments	Thickness with ornamentation in pole	Thickness with ornamentation in equator	Thickness without ornamentation in pole	Thickness without ornamentation in equator	Pore density in pole	Pore density in equator
31°	0.57 ± 0.13	0.66 ± 0.09	0.30 ± 0.07	0.34 ± 0.07	0.17 ± 0.21	0.19 ± 0.21
33°	0.50 ± 0.15	0.67 ± 0.09	0.30 ± 0.05	0.35 ± 0.04	0.12 ± 0.16	0.31 ± 0.24
9 hs 33°	0.56 ± 0.12	0.69 ± 0.09	0.31 ± 0.06	0.35 ± 0.06	0.19 ± 0.23	0.46 ± 0.70
15 hs 33°	0.54 ± 0.11	0.67 ± 0.10	0.31 ± 0.04	0.35 ± 0.05	0.29 ± 0.49	0.51 ± 0.66

## Discussion

Through the magnifying glass and SEM, we were able to distinguish structures already reported in previous studies for *C. latirostris*, such as the calcite layer crossed by pores and remains of protein membrane^3,6,7^. We also observed that eggshells had an ornamentation, made up of columnar structures, some of them joined by bridges (Fig. 2), depending both on erosion by external factors during incubation and on the characteristics of the nest itself^6,7,16^.

We found structures, which we called “intracascaral space” (Figs. 1, 2, 3), in both pole and equator regions, in eggshells belonging to different nests from both captivity and wild, so we discard the idea that it is a feature produced only by a single female, or because of captivity. Moreover, we have observed “intracascaral space” in eggshells of freshly deposited eggs, fertile and infertile eggs, which would be evidence that such structures would not be the result of structural modifications during incubation, nor defects related to the egg infertility.

The origin of “intracascaral space” could be explained by the absence or non-deposition of crystals in that section, creating that empty space during egg formation. Descriptions of the formation process of bird eggs (Archosauria), mention that a protein matrix is created on the surface of the testaceous membrane that will regulate the mineralization of the eggshell. The proteins secreted by female’s oviduct are part of this matrix and participate in the establishment of the calcite crystals^28^. Therefore, we speculate that during eggshell formation the sites where these “intracascaral space” are formed would be high in protein concentration, thus inhibiting calcium deposition.

We do not know their function, although we assume they may be involved in the formation of pore channels. This hypothesis is based on the fact that these structures are found below the craters (from the outside) and above the pore channels (from the inside); the “intracascaral space” are located near the outer surface. We could observe one or several “intracascaral space”, stacked on top of each other (Figs. 4, 5). Our idea is that these intracascaral space could constitute lines of fragility or points of fracture, whose function would be of structural change, since they would facilitate the opening of pore.

Once the whole system is connected (pore channel and intracascaral space, connecting outside and inside of the egg), another function that intracascaral space could have is air reserve in case the nest is flooded. Indeed, it was reported that *C. latirostris* eggs have other structures that could have evolved to survive nests flooding^3^.

This is the first report on this new structure found in *C. latirostris* eggshells, and we do not know if there are “intracascaral space” in the eggshells of other crocodile species. Therefore, in the present study we present hypotheses about the possible origin and functions according to previous studies and evidences.
